# Implementing falls prevention patient education in hospitals - older people’s views on barriers and enablers

**DOI:** 10.1186/s12912-024-02289-x

**Published:** 2024-09-11

**Authors:** Anne-Marie Hill, J. Francis-Coad, S. Vaz, M. E. Morris, L. Flicker, T. Weselman, J. A. Hang

**Affiliations:** 1 School of Allied Health, University of Western Australia, 35 Stirling Highway, Crawley, Perth, WA 6009 Australia; 2 WA Centre for Health & Ageing, University of Western Australia, Perth, WA Australia; 3 Ngangk Yira Institute for Change, Murdoch University Western Australia, Murdoch, Australia; 4 Academic and Research Collaborative in Health and Care Economy Research Institute, La Trobe University, Melbourne, VIC Australia; 5 Victorian Rehabilitation Centre, Glen Waverley, Melbourne, VIC Australia

**Keywords:** Consumer engagement, Falls prevention, Accidental falls, Patient education, Patient experience, Older adults

## Abstract

**Background:**

World falls guidelines recommend that hospitalised older patients receive individualised falls prevention education, yet no studies have sought older people’s feedback on how best to deliver falls prevention education in hospitals. The objective of the study was to explore the perspectives of older people and their caregivers about barriers and enablers to implementation of a tailored hospital falls education program.

**Methods:**

A qualitative descriptive design was used. Three focus groups and 16 semi-structured interviews were conducted. A purposive sample of older people who had previous hospital admissions and caregivers of older people were selected to review a co-designed patient falls education program (the revised Safe Recovery program). They provided feedback on how to implement the program in hospital settings. Data were thematically analysed taking an deductive-inductive approach.

**Results:**

Participants were 37 older people [female *n* = 24 (64.9%), age range 64 to 89 years] and nine caregivers (female *n* = 8). The first theme was that the Safe Recovery Program resources were of high quality, enabling strong patient engagement and increased knowledge and awareness about falls prevention in hospitals. The second theme identified practical strategies to enable program delivery in hospital wards. The key enablers identified were: timing of delivery around wellness and the patient’s mobility; tailoring messages for each older patient; key staff members being assigned to lead program delivery. Participants recommended that staff assist older patients to set appropriate behavioural goals in relation to preventing falls in hospitals. They also recommended that staff raise older patients’ confidence and motivation to take action to reduce the risk of falls. Providing resources in other languages and alternative shorter versions was recommended to enable broad dissemination.

**Conclusions:**

Older people and their caregivers advised that implementing falls education in hospitals can be enabled by using high quality resources, delivering falls education in a timely manner and personalising the education and support to individual needs.

**Supplementary Information:**

The online version contains supplementary material available at 10.1186/s12912-024-02289-x.

## Background

Falls sustained by older patients in hospital frequently cause physical injury, including fracture and head injuries [1–3]. These falls are associated with negative impacts on health, including adverse social and psychological outcomes [4–6] and incur a significant financial cost to healthcare systems [7, 8]. Recent world falls guidelines recommend educating patients about falls prevention to reduce falls and associated injuries whilst in hospital [9]. Evidence from randomised controlled trials (RCT) and meta-analyses demonstrates that falls and falls injuries can be reduced with patient education alongside supportive staff training [2, 10–12].

Despite the benefits of patient education for reducing hospital falls, [9, 12] there is scant evidence of systematic implementation of patient education [13]. Barriers identified to providing timely patient education include limited interprofessional communication about falls, sub-optimal hospital policies and systems for falls education, and staff perceptions of patient-related barriers to receiving falls education [14]. Health professionals have identified the need to overcome organisational, patient and clinician-related barriers to effectively implement falls education whilst older people are in hospital [15]. There are limited educational materials tailored for older people and the depth and breadth of research in this area is lacking [16]. Older people’s needs should be directly addressed when providing health education, including providing age-appropriate educational resources with relevant images and large text [16, 17].

Previous studies have investigated the perspectives of older patients who have fallen and also evaluated older patients’ perceptions of the risk of falling while in hospital and their views about receiving hospital falls education [18–21]. However, although hospital staff have been consulted about how to provide falls prevention education, no study has consulted with older people, who are the ‘consumers’ who receive this education, to understand their perspectives about implementing falls prevention education programs in hospitals. Obtaining consumer (older people’s) feedback is an important consideration for successful implementation because individual consumer characteristics, such as knowledge and attitudes, engender either positive or negative responses to an intervention and hence impact strongly on whether it is adopted [22, 23].

The Safe Recovery fall prevention education Program (SRP) was developed to educate older patients about keeping safe while in hospital [24, 25]. Two previous multi-site RCTs (in 2012 and 2015) support the effectiveness of the SRP for reducing falls and injuries [2, 26]. SRP messaging is grounded in health behaviour change theory, which applied to falls prevention suggests that when an older person develops risk awareness and knowledge about falls prevention, motivation to take action increases with social and environmental opportunity [27]. Recently, Francis-Coad et al., (2023) reported the development and evaluation of a revised SRP. The revised SRP consists of two components: a multimedia education package (which comprises of a video and a written guidebook); and a series of individual follow-up sessions with a staff member. The revised version was created using a participatory approach [24] and older patients who received the revised SRP demonstrated significant improvements in knowledge, awareness, motivation and intention to reduce their risk of falling [24]. Since both staff and patients were highly positive about the revised program [24] it was planned to implement it more broadly into other wards and settings.

However, clinical practice guidelines provide inconsistent and limited recommendations about how to systematically implement patient and family education such as the SRP [13]. Recently, we explored older consumers and their caregivers’ perspectives about falls prevention education provided to them when hospitalised [28]. These older people advised they were unsure about how and when to take action while in hospital to reduce their risk of falls. They also reported that falls prevention education they received was insufficient and inconsistent and not always personalised to their needs [28].

Given the paucity of implementation research for delivering patient falls education, we first sought to obtain older people’s feedback to inform implementation of the revised SRP. The objective of the study was to explore older people and their caregivers’ perspectives about barriers and enablers to implementing the revised Safe Recovery Program in hospitals.

## Methods

### Design

The study used a qualitative description design [29]. Qualitative description aims to stay close to participants’ experiences and focus groups and semi-structured interviews were used to facilitate exploration of individuals’ experiences with depth and rigor [30]. This allowed analysis of the data to capture a rich description of older people’s practical feedback on barriers and enablers to implementation of patient fall prevention education programs in hospitals [29, 31].

### Setting

Participants were recruited in Western Australia (WA), where hospital care is provided in settings that include large, tertiary or secondary hospitals and regional and small rural hospitals. Systematic falls prevention programs operate throughout WA hospitals as part of national health care regulations for safety and quality assurance. These underpin admission and ward procedures for all patients.

### Participants

A purposive sample of older people was used to ensure a diverse range of participant characteristics, including types of hospital experiences, age, gender and health status. Inclusion criteria included being 65 years or older and having experience of being an inpatient in hospital. All hospital experiences, either medical or surgical and any length of stay were considered valuable, as it would broadly inform an older person’s feedback about how hospitals can implement effective patient falls education. Participants needed to be able to provide informed consent and communicate in English. Caregivers of older people were also eligible for inclusion if they could share experiences of the older person they cared for being admitted to hospital.

### Recruitment

The research team collaborated with a WA not-for-profit organisation dedicated to preventing falls injuries in the community for 30 years [32]. This organisation interacts with groups of older people in the community on a regular basis providing information and education about falls prevention. The organisation referred older people who met the inclusion criteria (of age and experience being in a hospital), or caregivers who cared for an older person, to the research team. The researchers screened potential participants to confirm they met the purposive sample inclusion criteria. Subsequently, further screening was undertaken to address the sample variation requirements. Eligible participants were then presented with verbal and written information about what participation in the study entailed and invited to provide informed consent. All older adults and caregivers who were invited agreed to participate in the study.

### Data collection

Participants could choose to participate in either a focus group or a one-on-one semi structured interview. These options were provided to accommodate individual preferences and address potential apprehensions about attending face to face sessions, particularly in light of infectious disease concerns, for example COVID19. The focus groups or interviews were used firstly to gather information about any prior falls education participants had received in hospital [28]. Following this discussion, participants proceeded to review the revised SRP. At the commencement of each focus group or interview, participants were administered a brief survey to collect demographic data and the researchers explained the purpose of the research. Focus groups were held at a local community centre and interviews were either conducted in person at a private room at a community venue, or online via the Microsoft Teams application (Version 1.6.00.11166), with all interviews and focus groups being audio-recorded. The focus groups involved three to ten participants, lasted two to three hours and were facilitated by two to three researchers (AMH, TW, SV) who shared responsibilities for moderation, note taking and observation. One researcher (TW), supported by AMH, conducted individual semi-structured interviews either in person or online, with each interview taking approximately 45 to 60 min. Member checking was performed at the end of each focus group or interview to add credibility and confirmability to the findings [30]. The research team was comprised of experienced health professionals (physiotherapists, occupational therapist, social worker, geriatrician) who had completed research training and accumulated over 15 years of direct experience working with older individuals in research. Continuous collaboration amongst the researchers ensured a comprehensive understanding of the data, and data collection concluded when researchers agreed that no new variants of data were emerging.

### Topic guide

At the beginning of the second half of the focus group or interview, participants reviewed the revised SRP (video and workbook). Participants had not previously been exposed to the SRP and were first provided with a short summary of how the program was delivered. Participants were informed that all feedback would be valuable for developing the implementation plan. The Safe Recovery Program video was shown twice and participants were given time to read through the guide book. Segments of the video were re-watched by participants as desired during the discussion and participants were given time to browse both guide book and video content. The session used a discussion guide (see supplementary file 1) that directed the conversations.

### Data analysis

Descriptive statistics were used to summarise the sample demographic profile. Qualitative data were transcribed verbatim and managed using NVivo (QSR International Pty Ltd). To ensure accuracy and completeness, two researchers (TW, AMH) manually verified transcriptions [33]. A deductive-inductive approach was used to code the data, using reflexive thematic analysis [34, 35]. The deductive approach allowed for testing of responses against a framework of identifying barriers and enablers to implementing the revised SRP in hospitals. An inductive approach was then applied to explored unanticipated responses. The six-step process consisted of: gaining familiarity with the data set with repeated reading of transcripts and listening to audio files, generating initial codes prior to generating initial candidate themes, (and producing a ‘thematic map’), and subsequently defining and naming the final themes, and producing the analytical report. Researchers (TW and AMH) independently coded an initial set of transcripts before working collaboratively to develop potential themes. A back-and-forth approach and discussions with a third senior researcher (JFC) throughout the analysis ensured trustworthiness of the findings. The consolidated criteria for reporting qualitative research (COREQ) guidelines were followed (see supplementary file 2) to improve the quality and transparency of the reporting [36].

## Results

### Participant characteristics

There were 46 participants (37 older people and 9 caregivers). Sixteen older people completed an individual semi-structured interview and remaining participants joined one of three focus groups. The characteristics of the sample have been described previously [28] and are presented in Table 1. Participants (*n* = 24 female, 13 male older adults) had a range of functional ability, with 11(29.7%) using a walking aid, 13 (35.2%) able to do limited or no tasks around the house unless they had assistance, 25 (67.6%) had a history of falls and 11 (29.7%) reported a ‘near miss’ where they almost fell. All older people had experiences of being an inpatient in a hospital. Over 70% of the older people had been admitted to a large tertiary hospital, and over 55% had more than one hospital admission. Older people’s median length of stay during their hospital admission was 4.5 days (interquartile range = 8 days).

**Table 1:** Participant characteristics

**a. older participants’ characteristics**			
Characteristics	Total *n* = 37 (100%)	Focus Groups *n* = 22 (100%)	Personal Interviews *n* = 15 (100%)
Gender, female	24 (64.9)	16 (72.7)	8 (53.3)
Age range			
60–64	1 (2.7)	0 (0.0)	1 (6.7)
65–69	4 (10.8)	2 (9.1)	2 (13.3)
70–74	9 (24.3)	3 (13.6)	6 (40.0)
75–79	14 (37.9)	9 (41.0)	5 (33.3)
80–84	5 (13.5)	5 (22.7)	0 (0.0)
85–89	4 (10.8)	3 (13.6)	1 (6.7)
Highest level of education^a^			
High school	12 (32.4)	9 (40.9)	3 (20.0)
Technical collage	15 (40.5)	9 (40.9)	6 (40.0)
University	7 (18.9)	2 (9.1)	5 (33.3)
Vision and hearing^a^			
Good vision and hearing	13 (35.1)	10 (45.4)	3 (20.0)
Poor vision only	13 (35.1)	2 (9.1)	11 (73.3)
Poor hearing only	2 (5.4)	2 (9.1)	0 (0.0)
Poor vision and hearing	7 (19.0)	6 (27.3)	1 (6.7)
Number of prescribed medications^a^			
Less than 4	18 (48.7)	10 (45.5)	8 (53.3)
4 to 8	12 (32.4)	8 (36.4)	4 (26.7)
9 or more	6 (16.2)	3 (13.6)	3 (20.0)
Using a walking aid, yes^a^	11 (29.7)	6 (27.3)	5 (33.3)
Walking frame	4 (10.8)	1 (4.5)	3 (20.0)
Walking stick	4 (10.8)	3 (13.6)	1 (6.7)
Scooter and quad stick	1 (2.7)	1 (4.5)	0 (0.0)
Ability to do jobs around the house			
Quickly and efficiently, without assistance	6 (16.2)	5 (22.7)	1 (6.7)
Relatively easily without assistance	10 (27.0)	5 (22.7)	5 (33.3)
Slowly without assistance	8 (21.6)	3 (13.7)	5 (33.3)
Limited tasks without assistance	9 (24.4)	5 (22.7)	4 (26.7)
Unable to complete tasks by myself	4 (10.8)	4 (18.2)	0 (0.0)
Hospital falls history			
Fell in hospital	3 (8.1)	1 (4.5)	2 (13.3)
Near miss fall in hospital	4 (10.8)	3 (13.6)	1 (6.7)
Overall falls history			
Fell at home after discharge from a hospital admission	7 (18.9)	4(18.3)	3 (20.0)
Previously fallen at home	25 (67.6)	14 (63.6)	11 (73.3)
Near miss fall at home	11 (29.7)	9 (40.9)	2 (13.3)
Hospital admission last 2 years			
1	17 (45.9)	10 (45.5)	7 (46.6)
2	12 (32.5)	8 (36.4)	4 (26.7)
3 or more	8 (21.6)	4 (18.1)	4 (26.7)
Type of hospital			
Tertiary	26 (70.3)	17 (77.3)	9 (60.0)
Secondary (included rehabilitation settings)	6 (16.2)	2 (9.2)	4 (26.6)
Other (included rural)	2 (5.4)	1 (4.5)	1 (6.7)
Several different hospitals	2 (5.4)	1 (4.5)	1 (6.7)
Another state of Australia	1 (2.7)	1 (4.5)	0 (0.0)
Hospital admission included surgery	23 (62.2)	16 (72.7)	7 (46.7)
Length of stay in hospital, median, (IQR)	4.5 (8)	4.4 (6)	6 (7.5)
Length of stay in hospital			
Overnight	8 (21.6)	4 (18.3)	4 (26.6)
1 night to a week	18 (48.6)	12 (54.5)	7 (40.0)
1 to 2 weeks	8 (21.6)	5 (22.7)	3 (20.0)
2 to ≥ 4 weeks	3 (8.2)	1 (4.5)	2 (13.4)
**b. Caregiver sample-characteristics**			
**Characteristics**		***n*** ** = 9** ^**b**^	
Age, years, range			
less than 54		4	
55–59		2	
60–64		1	
≥65		2	
Gender, female		8 (88.8%)	
Person caring for:			
Daughter caring for mother		4	
Daughter caring for father		3	
Husband caring for wife		1	
Caring for more than one person^c^		1	
Care recipient hospital admissions last 2 years			
One admission		1	
More than one admission		8	

a: where numbers are not *n* = 100%, participant did not respond to demographic survey item

*Notes* b: Eight carers attended focus groups, one was interviewed; c: An Aboriginal person who cared for multiple people in their community

### Theme 1. Positive reaction to program

Participants reacted positively to both the Safe Recovery Program video and guidebook. The high quality of these resources and the clarity of the information were viewed as enabling implementation because they promoted engagement with the content and subsequent learning about hospital falls and their prevention.

#### Sub theme. High quality program enables high level of engagement

The high-quality presentation of the revised SRP video resource was effective in capturing older people’s attention and communicating essential information.

“It’s very well presented…” [participant (P) 16, male]

The clear, straightforward language in the video was also noted as important in conveying understanding of the messages.

“I think it’s (video) very, very good. It’s clear….the language is spot on…very informative” (P10, female caregiver)

Older people viewed the revised SRP guidebook as complementary to the video and liked having easy access to a high-quality, tangible resource at their fingertips. Feedback highlighted the guidebook’s clarity, simplicity and readability.

“It was a great little handbook…especially like the pictures as they made the message so clear” (P16, male),

“That’s good [referring to the guidebook] because it’s written out very simply” (P41, female)

A few participants noted the length of the resources could be problematic for some older patients and could potentially overload them with information. They suggested the video length could be reduced with less repetition of the messages, to ensure patients reviewed all the essential content. Others suggested the guidebook could be shortened or adapted.

“Maybe just a bit too long…just a bit repetitive…” (P13, female)

“Another [leaflet or wall poster could be] way of getting the message across because there’s a lot of information” (P5, male)

#### Sub theme. Messages raise patients’ knowledge and awareness of falls and falls prevention

All participants reported gaining knowledge after viewing the program resources and appreciated how the program summarised the key learning in 3-succinct take-home dot points which were impactful and easy to remember. Participants reported being much more aware of their falls risk after viewing the program resources.

“Three points are good because people will retain quite a lot of that” (P46, female)

“It definitely made me aware…few patients will think they will fall over, that was me until I started reading this [guidebook] …” (P46, female)

Other participants emphasised the importance of raising awareness of falls risk among hospitalised older people. This included raising awareness of personal falls risk for older people who had either judged themselves as being at low risk of falling or those who had not sustained a fall. Being independent prior to hospitalisation was perceived as a potential barrier to taking up program messages.

“I think the first thing is to make a patient aware that there’s a high risk of falls…and a high risk of injury [if you fall]…therefore, that should be a reason for watching the video” (P5, male)

“Most people, myself included, we think we’re tougher than we are you know…being in hospital, you think you’re fine, you know” (P33, male)

#### Sub theme. Older patients need confidence and motivation to take action to prevent falls

Assistance to raise an older person’s motivation to enact the suggested behaviours was viewed as an important enabler to effectively implement the education and reduce hospital falls. Most participants strongly identified with the message of needing to ask for help but felt uneasy about initiating requests for assistance by ringing their bell. A gender stereotype difference was identified with men thought to be especially reluctant to seek help.

“There’s a huge part of the population that won’t use the bell…to call for help… whilst the physical presence of the bell is one part, the other part is the education [to use the bell]” (P36 female, caregiver)

“Reassurance from the nurses to say that…you know you’re not gonna be in trouble [if you ring the bell]…you’re gonna be more trouble if I find you on the floor” (P29, female)

“You know, men in particular…I suspect they’re the biggest challenge…men never ask for help unless their arms have been cut off!” (P34, female)

### Theme 2. Practical suggestions to aid falls education delivery

Participants made several practical suggestions about how staff could address possible barriers to effective implementation of the education program, to enable the older patient to receive the program and take action. Timing of program delivery, consistent and personalised communication and assistance with goal setting that could promote action by the older patient was highlighted by participants.

#### Sub theme. Right timing of program delivery

The physical and cognitive conditions of hospitalised older patients, such as pain, cognitive impairments, and the effects of medication, were felt to significantly impact on their ability to participate in, and benefit, from educational programs. Older people reflected that being preoccupied with medical issues while on the ward could increase anxiety and reduce the ability to concentrate on other things.

“When you go there at first…you’re in pain. You don’t want to know about these things. You want to be looked after” (P15, female)

“If I was in that position, I wouldn’t want to really listen to someone cause I’m not feeling the best…they have to give this information at a time that the person is receptive…” (P10, female caregiver)

“Pain, incontinence [distractions], trouble concentrating… can be worrying” (P16, male)

However, participants were cognisant of falls risk and their need for education. Suggestions to enable successful program delivery included timing the education delivery to when patients were able to engage, but prior to them undertaking activity.

“Best time to do it would be when they first come in, especially if they are a falls risk” (P11, female)

“When they’re recovering … Not, not, you know, if they’re coming out of anaesthetic or anything. But, you know, the few days before [they start moving]… in the middle, you know” (P9, female)

Participants’ viewpoints were that nursing staff being too busy and overworked could form a barrier to delivering timely education sessions to older people. The busy nature of hospital wards with patients receiving multiple care interactions and treatments was identified as a barrier to effective program delivery.

“They haven’t got the time. They’re too busy being nurses, you know” (P45, male)

“Because nurses have got a lot on their minds…And although it’s important that you reduce the risk of falling over, I think it’s probably more important they [nurses] get all the medications into all the patients at the right time” (P5, male)

“I think delivering it on the ward is the biggest barrier is I’m not sure that patients would be focused” (P12, male)

#### Sub theme. Effective communication enables better implementation

Participant feedback strongly focused on the importance of effective communication to minimise barriers to implementation of the program and take up of messages by the older patient. Older people highlighted the importance of appointing a dedicated staff member who would take a one-to-one approach in a fall prevention educator role. Personalised delivery was viewed as best to ensure the program’s effective implementation, implying that without such individual attention, materials might not be fully engaged with or understood.

“If they’re not going to do that it’s [program resources] of little use cuz it won’t be read, it won’t be looked at, won’t be understood possibly” (P26, female)

“It’s not enough just to put this on the shelf with all the other booklets - you need to personalise it!” (P37 female, caregiver)

Most older people suggested an allied health professional would be best suited, as their skill set could enable tailored communication and program adaptation, reflecting a patient-centred approach. Allied health professionals leading program delivery was additionally seen as advantageous because participants felt delivering the program could burden nursing staff. A few older people expressed another viewpoint, suggesting that anyone knowledgeable about the program could deliver it, provided they were not overwhelmed.

“I think in that context…a physio [physiotherapist]…would probably be the best person…to deliver it. Physio or OT [occupational therapist]” (P32, male)

“So it isn’t another demand on the nursing staff” (P34, female)

“A great book for the therapy assistant to work through” (P33, male)

Regarding communication, participants suggested that the educator should use a conversational style and a didactic program delivery style was viewed as a barrier to engagement.

“It shouldn’t be a lecture, but a conversation” (P16, male)

“Well I don’t like being told” (P4, male)

Participants strongly concurred that consistent communication from staff was an important enabler.

“The patient would have this booklet with themselves…how could we ensure that the nurse knows what goals are set…the physio knows what goals are set and other therapists and the doctor knows” (P39, female)

Several participants felt that having access to interpreters or having the resources translated to other languages would be an enabler to good communication for older people from culturally and linguistically diverse backgrounds, to assist them to better engage in the program.

“Non-English speakers will be less likely to comprehend the whole thing. So, I guess the other thing is the availability of interpreters, or whether it’s available in languages other than English” (P12, male)

#### Sub theme. Provide assistance with goal setting regarding falls prevention

Many older people reported the concept of goal-setting as unfamiliar and complex. This was a potential barrier to successful implementation of interventions that require active patient engagement in setting behavioural goals. Some participants explained they did not identify with the word ‘goal,’ citing generational differences. A recommendation was to use an alternative word to goal setting as this may be perceived more positively.

“The goals because I, for one, didn’t know anything about goals…It’s something that I’ve never even had any idea about…” (P4, male)

“Goal targeting to me is for the younger generation…you know people of school age that type of thing. When you get to older people, they’re not really up with all the kind stuff…” (P15, female)

“May be teasing out…what do you want to get out of this? But don’t use the word goal. I would do it, you know [by saying]…where do you want to get to?” (P4, male)

Further recommendations included the need for hospital staff to provide one-to-one sessions to guide older people with goal setting and expectations regarding strategies for falls prevention during their hospital stay.

“I think it’s great if someone sits with you and works your goals with you. Cause sometimes you do need someone to guide you, not to tell you what your goals are cuz they’re your goals…You wanna be able to get to the corridor on your own to guide them put into practice” (P38, female caregiver)

Other participants added that writing down their goals would be a challenge and felt that staff should be encouraging and supportive regarding patient goals.

“I would probably give it a bit of thought but…no, I wouldn’t write things down…not many would write” (P28, male)

“If you have goals, then, you need someone to pat you on the back” (P14, male)

## Discussion

In this study, older people and their caregivers provided valuable feedback for hospital staff about how to effectively implement a falls prevention education program in hospitals. These findings are timely, given that recent falls prevention guidelines recommend that individualised patient education be provided to all older people in hospitals to reduce the persistently high falls rates world-wide [3, 4, 9]. Only a handful of studies have explored older people’s views about hospital falls education [19, 37, 38]. None of these studies sought older people’s views and perspectives about how to implement hospital falls education.

Overall, older people and their caregivers concurred that the falls prevention education program they reviewed (the revised SRP) [24] was of high quality and raised their knowledge and awareness about falls prevention with succinct, impactful messages. Well-designed fall education programs typically improve patient knowledge and perception of risk and empower patients to engage in risk-minimising behaviours [19, 39]. Some participants, particularly older people identifying as male, highlighted the importance of raising risk awareness. They reflected that they retained a perspective of being at low risk of falls, even when feeling unwell. These older people’s reflections and insights strongly concur with previous findings that older patients consistently misjudge their falls risk as low rather than high [18, 40]. Recent reviews suggest that addressing this misconception is a critical enabler to effective implementation when nursing staff are seeking to promote safe patient behaviour around falls prevention [41, 42].

Older people enjoyed the high quality of the revised SRP resources which they felt promoted engagement with the intended messages, concurring with findings from the participatory trial that established the revised SRP [24]. Both the original and revised SRP use educational theory of adult learning and behavioural change principles [27, 43, 44]. Reviews have concluded that videos are more effective in improving health literacy than standard education and that written materials and audio-visual materials are most preferred by older patients [16, 45]. Older people and their caregivers’ positive affirmation of the resources suggests that staff could initiate falls education with multimedia resources prior to individualised follow up with the older patient to ensure that relevant messages that target individual risk factors are adopted. Some older people observed that nursing staff were busy and suggested receiving support from other health professionals. Participants’ reflections may indicate that staff need to inform patients regularly about the role of the multidisciplinary team such that patients feel confident that all team members can work together to assist them. Recent evidence on care workforce redesign shows that other members of the multi-disciplinary team and healthcare assistants can share the load of delivering patient falls education in hospitals [11, 46].

Participants, many of whom had multiple prior hospital admissions, provided clear feedback about enablers to implementation from an older person’s perspective. Key enablers were articulated as taking a personalised approach to the education, timely delivery according to the individual patient’s stage of recovery and building patients’ confidence and motivation to seek help. Using a personalised approach to delivery and personalising the messages was seen as critical for ensuring messages aligned with the patients’ current health and mobility profile and their ability to comprehend and engage with the material. Our participants’ perspectives independently concurred with interventions delivered in RCT that have demonstrated reductions in falls and injurious falls. In these effective trials, ward staff provided patients with falls information and extended this education to engaging patients in their own falls risk assessment and development of a falls prevention plan [2, 10, 11]. Timing falls education to be early in admission and accommodate the varied states of receptiveness and health conditions also accords with these successful interventions [2, 10, 11]. Participants suggested creating brief versions of the resources that delivered essential components of the messages to cope with older patients either being distracted by the ward procedures or feeling unable to concentrate.

Participants emphasised that they needed help with developing goals for falls prevention, which concurs with previous findings identifying how hospital staff need to assist with goal setting and provide falls information [47, 48]. They also emphasised the need for support from staff to be confident to enact goals such as asking for assistance. A strong recurring theme throughout global research focused on in-hospital falls is that older patients, particularly male patients, feel reluctant to ask staff for help [18–21, 37, 49]. This highlights the need for ward staff to provide each older patient with positive support to engage in safe behaviours. Participants’ perspectives aligned with the Capability-Opportunity-Motivation-Behaviour (COM-B) framework of health behaviour change [27] whereby participants reflected that the education program raised their ‘Capability’ (knowledge and awareness), but they identified that they required help, particularly from nursing staff, to build their ‘Motivation’ (confidence to ask for help) and provide ‘Opportunity’ for them to enact recommended falls prevention ‘Behaviours.’

The patient engagement that underpinned this study provides further feedback that can assist to promote effective implementation of evidence-based falls education in hospitals. Patient engagement is an essential pillar to improve the quality of health care and the level of engagement can influence the impact on services [23]. Engaging with patients can improve patients experiences and promote positive feelings of empowerment and hope [50].

### Strengths and limitations

Trustworthiness was aided by using member checking and researcher triangulation of data analysis and the researchers’ use of an audit trail [31]. A limitation of the study was that the research was conducted within one health setting. The researchers aimed to provide a rich data description to allow other health professionals to judge the extent of the transferability of the results to their own setting [51]. A strength of the study was that the sample was drawn from older people who were not current patients in hospitals and the focus groups were led by independent researchers who did not work in hospitals. This aimed to ensure firstly, that we sought meaningful feedback from our intended ‘consumers’ (older people and their caregivers) prior to implementing the program [23] and secondly, that older people were confident to provide independent feedback and advice since they were not in a healthcare relationship with the hospitals or the researchers [30, 52]. A limitation was that participants were not exposed to the program in a hospital environment. In our previous trial, older people who were in hospital and received the revised SRP as part of their admission provided positive feedback about program acceptability [24]. In the next phase of our research, we plan to implement the revised SRP on wards and seek further feedback from older patients. The revised SRP was reviewed by older people from English speaking backgrounds and participants recommended that resources be translated into other languages. We were unable to recruit older adults from culturally diverse backgrounds. We plan to undertake further research to translate the program into other languages and ask older people from culturally diverse backgrounds to provide feedback about implementation. Older people with a diagnosed cognitive impairment were not part of the sample, although some caregivers provided support to older people with cognitive problems. These older people are known to be at higher risk of falls in hospital and further research to discuss how to provide clear education for these older people and their families is required.

## Conclusion

Falls in hospitals continue to cause injury particularly in older patients. Older people and their caregivers identified that high quality falls education resources promoted engagement with falls prevention messages in hospitals wards. Older people requested that hospital staff deliver education in a timely manner, personalise messages to individual needs and raise confidence and motivation in older patients to undertake falls prevention action.

## Electronic supplementary material

Below is the link to the electronic supplementary material.

Supplementary Material 1

Supplementary Material 2

## Data Availability

The datasets used and analysed during the current study are available from the corresponding author on reasonable request.
