# Supplementary material for: Implementing falls prevention patient education in hospitals - older people’s views on barriers and enablers
Source: BMC Nurs. 2024 Sep 11;23:633. doi: 10.1186/s12912-024-02289-x (PMC11389421; doi:10.1186/s12912-024-02289-x)
Supplement: Supplementary file 1 — Supplementary Material 1 [file 12912_2024_2289_MOESM1_ESM.pdf]

Supplementary file 1. Interview Discussion Guide

Viewing the Revised Safe Recovery Program's resources and reflecting on the material (video+workbook)

Probing Qs

- What did you think about the Safe Recovery Program's video and workbook?
- What was helpful about it?
- What was not so helpful about it?
- Thinking about your own or a family member's last hospital stay
  - Do you think the content would have been relevant to your hospital stay?
  - Do you think you / their relative would respond/ enact messages?
  - Would you have felt any more or less safer after receiving this program?

- How do you see this working in a hospital ward?
- Would you do the mobility goal setting?
- Who do you think would be the best to support you in setting a mobility goal? (Explore: nurse, doctor, health professional, family member)
- What would be the best way to do it?
- Is there anything you would change about the program to make it better?

Brainstorm how and where the messages might be delivered to older people during or before hospital admission and ways to support health professionals in delivering education about falls to older patients in hospital

Probing Qs

- The barriers and enablers to implementing fall prevention education for patients in the hospital setting
- Brainstorm how and where the messages might be delivered to older people during or before hospital admission and ways to support nurses and health professionals in delivering the Safe Recovery Program resources to older patients in hospitals
  - Prompts: What if you are feeling sick?… tired?….confused? Should family members be involved?

Large group discussion, group member-checking and closing the community discussion/interview
