# Supplementary material for: Implementing falls prevention patient education in hospitals - older people’s views on barriers and enablers
Source: BMC Nurs. 2024 Sep 11;23:633. doi: 10.1186/s12912-024-02289-x (PMC11389421; doi:10.1186/s12912-024-02289-x)
Supplement: Supplementary file 2 — Supplementary Material 2 [file 12912_2024_2289_MOESM2_ESM.pdf]

Supplementary file 3.

Consolidated criteria for reporting qualitative studies (COREQ): 32-item checklist

For manuscript: Hill AM, et al. Implementing falls prevention patient education in hospitals - older peoples' views on barriers and enablers

No. Item	Guide questions/description	Reported on Page #
**Domain 1: Research team and reflexivity**		
Personal Characteristics		
1. Inter viewer/facilitator	Which author/s conducted the interview or focus group?	8
2. Credentials	What were the researcher's credentials? E.g. PhD, MD	No place to report other than title page – author details according to journal convention will be on the final published details on the journal publication page
3. Occupation	What was their occupation at the time of the study?	8 – under data collection
4. Gender	Was the researcher male or female?	n/a for this study
5. Experience and training	What experience or training did the researcher have?	8
Relationship with participants		
6. Relationship established	Was a relationship established prior to study commencement?	No
7. Participant knowledge of the interviewer	What did the participants know about the researcher? e.g. personal goals, reasons for doing the research	8-9, explained during the focus groups and interviews.
8. Interviewer characteristics	What characteristics were reported about the inter viewer/facilitator? e.g. Bias, assumptions, reasons and interests in the research topic	5, 6, Reasons for the research – last two paragraphs of introduction

**Domain 2: study design**		
Theoretical framework		
9. Methodological orientation and Theory	What methodological orientation was stated to underpin the study? e.g. grounded theory, discourse analysis, ethnography, phenomenology, content analysis	6, Design section
Participant selection		
10. Sampling	How were participants selected? e.g. purposive, convenience, consecutive, snowball	6, Participant section
11. Method of approach	How were participants approached? e.g. face-to-face, telephone, mail, email	7, Recruitment section
12. Sample size	How many participants were in the study?	9, Results section first paragraph
13. Non-participation	How many people refused to participate or dropped out? Reasons?	n/a
Setting		
14. Setting of data collection	Where was the data collected? e.g. home, clinic, workplace	6 and 8 setting and data collection sections
15. Presence of non-participants	Was anyone else present besides the participants and researchers?	(No) 8, data collection
16. Description of sample	What are the important characteristics of the sample? e.g. demographic data, date	10 & Table 1
Data collection		
17. Interview guide	Were questions, prompts, guides provided by the authors? Was it pilot tested?	See supplementary file 1
18. Repeat interviews	Were repeat inter views carried out? If yes, how many?	n/a
19. Audio/visual recording	Did the research use audio or visual recording to collect the data?	Yes , 8 data collection
20. Field notes	Were field notes made during and/or after the interview or focus group?	Yes , 8 data collection
21. Duration	What was the duration of the interviews or focus group?	8 data collection section
22. Data saturation	Was data saturation discussed?	8, data collection section
23. Transcripts returned	Were transcripts returned to participants for comment and/or correction?	No –8, member checking at time of interviews
**Domain 3: analysis and findings**		

Data analysis		
24. Number of data coders	How many data coders coded the data?	3 see page 9 analysis section
25. Description of the coding tree	Did authors provide a description of the coding tree?	9, (Six-phase guide to reflexive thematic analysis used)
26. Derivation of themes	Were themes identified in advance or derived from the data?	Derived from data
27. Software	What software, if applicable, was used to manage the data?	9, NVivo
28. Participant checking	Did participants provide feedback on the findings?	8 data collection section, through member checking at time of interview and focus groups
Reporting		
29. Quotations presented	Were participant quotations presented to illustrate the themes/findings? Was each quotation identified? e.g. participant number	Results section, 10 - 16
30. Data and findings consistent	Was there consistency between the data presented and the findings?	See themes in results section and discussion section congruence
31. Clarity of major themes	Were major themes clearly presented in the findings?	9, 13, two major themes named
32. Clarity of minor themes	Is there a description of diverse cases or discussion of minor themes?	10-16 see sub themes and divergence within each
